# “To know or not to know…?” Push and pull in ever smokers lung screening uptake decision‐making intentions

**DOI:** 10.1111/hex.12838

**Published:** 2018-10-05

**Authors:** Janet E. Tonge, Melanie Atack, Phil A. Crosbie, Phil V. Barber, Richard Booton, Denis Colligan

**Affiliations:** ^1^ Macmillan Cancer Improvement Partnership Parkway Business Centre Manchester Health and Care Commissioning Manchester UK; ^2^ North West Lung Centre, Wythenshawe Hospital Manchester University NHS Foundation Trust Manchester UK; ^3^ Present address: The Christie NHS Foundation Trust Manchester UK; ^4^ Present address: Leeds Institute of Health Sciences University of Leeds UK

**Keywords:** health literacy, informed participation, lung cancer, screening, smoking

## Abstract

**Background:**

In the United States, lung cancer screening aims to detect cancer early in nonsymptomatic current and former smokers. A lung screening pilot service in an area of high lung cancer incidence in the United Kingdom has been designed based on United States trial evidence. However, our understanding of acceptability and reasons for lung screening uptake or decline in a United Kingdom nontrial context are currently limited.

**Objective:**

To explore with ever smokers the acceptability of targeted lung screening and uptake decision‐making intentions.

**Design:**

Qualitative study using semistructured focus groups and inductive thematic analysis to explore acceptability and uptake decision‐making intentions with people of similar characteristics to lung screening eligible individuals.

**Setting and participants:**

Thirty‐three participants (22 ex‐smokers; 11 smokers) men and women, smokers and ex‐smokers, aged 50‐80 were recruited purposively from community and health settings in Manchester, England.

**Results:**

Lung screening was widely acceptable to participants. It was seen as offering reassurance about lung health or opportunity for early detection and treatment. Participant's desire to know about their lung health via screening was impacted by perceived benefits; emotions such as worry about a diagnosis and screening tests; practicalities such as accessibility; and smoking‐related issues including perceptions of individual risk and smoking stigma.

**Discussion:**

Decision making was multifaceted with indications that current smokers faced higher participation barriers than ex‐smokers. Reducing participation barriers through careful service design and provision of decision support information will be important in lung screening programmes to support informed consent and equitable uptake.

## 1 INTRODUCTION

Despite fewer people smoking, lung cancer causes nearly 36 000 deaths annually in the United Kingdom (UK)^1^ with incidence rates forecast to increase until 2030 due to the ageing population and risk reduction times following smoking quits.^2^ How quickly lung cancer is found is strongly linked to survival times.^3^ Just 17% of people diagnosed with stage IV lung cancer are alive 12 months later, compared to 83% diagnosed at stage I.^4^ Survival rates have not increased much over the last four decades^4^ and late‐stage diagnosis remains the experience of the majority.^1^ Into this gloomy picture has come the results of the National Lung Screening Trial (NLST)^5^ in the United States which reported 20% fewer lung cancer deaths following yearly screening with low‐dose computerized tomography (LDCT) over 3 years compared to chest X‐rays in 53 456 high risk ever smokers.^6^ This led to the introduction of lung cancer screening for 55‐ to 77‐year‐olds (if smoked for 30 pack‐years or more and were continuing or stopped within 15 years)^7^, ^8^ in the United States and calls for screening to be introduced elsewhere.^9^, ^10^, ^11^

Lung cancer screening is not currently approved as a UK wide programme,^12^ and questions have been asked about the balance of harms and benefits from being screened.^13^ The UK Lung Screening Study (UKLS), a pilot randomized control trial undertaken in the Merseyside and Cambridgeshire areas with smokers and ex‐smokers aged 50‐75, encouragingly reported 85.7% cancers were diagnosed at stages I or II in its screening arm, but was underpowered to detect mortality impact.^14^ UK national policy with regard to lung screening will be reviewed when European lung cancer screening trial results have been pooled.^15^ However, in the meantime funding for National Health Service (NHS) lung screening pilots have been announced.^16^

It has been suggested that as LDCT scans are quick, painless and identify treatable disease, screening should be publically acceptable, despite some radiation risk.^17^, ^18^, ^19^ Indications are that lung screening invitees perceive screening benefits to include gaining early treatment and relief from worry about having lung cancer but not all invitees wish to be screened.^20^, ^21^, ^22^ Lung screening is unique amongst cancer screening as it is looking for a disease closely linked with smoking which is strongly stigmatized.^18^ Lung screening uptake by smokers, often living in deprived areas, has been identified as a particular challenge.^23^, ^24^, ^25^, ^26^, ^27^ Patel et al^18^ categorized nonresponders to lung screening as “too old to be bothered,” “worriers,” “fatalists” or “avoiders”. Avoiding finding out if cancer was present, perceptions of personal risk, individual benefits, practical and emotional barriers have all been identified as influences in lung screening uptake decision making.^14^, ^21^, ^22^, ^28^ Other studies have reported that current smokers more than ex‐smokers consider cancer a “death sentence” expect less benefit from early detection and have higher uptake barriers.^15^, ^17^, ^22^, ^29^ Contrastingly, Cataldo^30^ reported that older smokers were receptive to lung screening and suggested that further understanding of smokers’ views was needed.^15^, ^17^, ^19^, ^22^, ^29^ Our understanding of the views of ever smokers about lung screening is currently limited in a UK nontrial context.^14^, ^18^ The only other lung screening study exploring the views of ever smokers in UK nontrial context which the authors are aware of found cancer fatalism, low lung health expectations and smoking stigma as uptake barriers.^19^

To test the viability of lung screening outside of a research trial context, an NHS pilot screening service has now taken place in Manchester, England.^31^ This offered a “lung health check” (risk assessment and spirometry) to current and former smokers aged 55‐74, with no lung cancer diagnosis within 5 years and who were not on a primary care palliative care register. Appointments were in mobile facilities in supermarket carparks. An LDCT scan was offered immediately on site for individuals with a 1.51% lung cancer risk or higher in the next 6 years unless they had a chest CT scan within 12 months. The Prostate, Lung, Colorectal and Ovarian Cancer Screening Trial risk model (PLCO_M2012_) was used to estimate individual risk.^32^ Invitation was by GP letter (not open access drop in) with explanatory information. Results were by letter or follow‐up hospital appointment.^31^ A follow‐up scan was offered 3 months after the first scan for individuals with indeterminate results. The first lung health checks and scanning round took place in June 2016 to February 2017. All individuals who had an initial scan were invited for a second scan 12 months later. Baseline results from the first scan round were that 80% of lung cancers were found at stages I and II. This was a significant stage change (*P* < 0.0001) compared to the same area the year before and allowed 89% of people where lung cancer was found to be offered curative intent treatment.^31^ Initial findings from this qualitative study (first four focus groups held February to May 2016) informed thinking about the Manchester pilot service design and its patient information materials.

## 2 OBJECTIVES

The study objectives were to explore with ever smokers aged 50‐80 in Manchester:

1. The acceptability of lung screening via a lung health check and LDCT scan
2. Influences on uptake intentions.

The main research question was:

Is a targeted approach to the early diagnosis of lung cancer acceptable in a high risk Manchester population?

Targeting refers to the lung screening eligibility criteria based on smoking status, smoking history and age. “Acceptability” was considered as positivity about the service in principle and expressed intent for use.

## 3 METHODS

### 3.1 Design, setting and participants

We undertook a qualitative study to explore lung screening acceptability and uptake decision making with people of similar characteristics to screening eligible individuals. Qualitative research is a recognized way to explore potential service users views^33^, ^34^, ^35^ and assist in translating research to real‐world practice.^36^ This is important as most lung screening knowledge is from experimental settings.^21^ The study setting was Manchester, a “postindustrial” city^37^ with significant deprivation, higher lung cancer incidence and lower life expectancies than England averages.^38^ The study team included members trained in qualitative techniques, with significant understanding of lung cancer and participant recruitment working within Manchester's NHS. In addition, wider academic expertise was sought to help design and support the study, including people who were able to provide relevant topic and methodological expertise.

Participant recruitment was undertaken in community venues and NHS premises. Inclusion criteria were men and women, current and former smokers, aged 50‐80 (to reflect variation across lung screening initiatives) who had not had lung cancer. Posters advertising the study were distributed widely by the Black Health Agency for Equality and Manchester Health and Care Commissioning. An information sheet explained the study and right to withdraw. Participants were recruited using purposive sampling. When a person expressed interest, inclusion criteria and characteristic criteria (gender, age, address deprivation, working status and ethnicity) were checked to enable participant selection and target recruitment on sample gaps. A £10 gift voucher was offered as participation thanks and travel expenses were refunded.

### 3.2 Data collection

Data collection used semistructured focus groups to allow participant interaction to generate understanding.^39^, ^40^ To facilitate freer expression and comparison, separate groups for smokers and ex‐smokers were held. Six groups (three for smokers and three for ex‐smokers) were held in accessible community locations (February to June 2016). It was anticipated that given the study scope, this would provide sufficient data without missing new insights.^41^ Each group was mixed by gender, age, ethnicity and address deprivation. The corresponding author acted as group moderator supported by an assistant moderator. The topic guide included lung screening, lung cancer beliefs and uptake intentions. Groups were audio‐recorded, transcribed verbatim and checked for accuracy. Participants were given service information and asked what they would do if they received a screening invite; responses were noted following a show of hands.

### 3.3 Analysis

To protect anonymity, pseudonyms were used and identifiable information in transcripts extracted before uploading into computer software N‐Vivo 11 with characteristic information and address deprivation from postcodes matched to Indices of Multiple Deprivation levels.^42^ Inductive thematic analysis^43^ was chosen to identify themes within data rather than use pre‐existing literature concepts.^44^ Analysis used Braun and Clarke's recommended steps for thematic analysis^43^ and was completed separately for smoking and ex‐smoking groups. This commenced with data familiarization (listening to recordings and rereading transcripts). Early codes were identified, added to transcript sections and reread to identify groups of similar comments by the corresponding author. A description of early categories was written and the data checked for correct inclusion by the corresponding author. Discussion about the categories was held between the corresponding author and assistant moderator. Initial themes^43^ were identified by the corresponding author who considered categorized data to see what patterns and linkages existed and discussed between an independent researcher and the corresponding author. Theme identification and interpretation were assisted by “writing memos”, “diagramming”^45^ and asking questions about significance, underpinning influences and implications.^43^ Participant characteristics were also considered to identify any patterns and linkages. Refinements were made by consensus until a final “thematic map” was considered appropriate.^43^ Theme saturation was considered to have taken place when additional data did not add new information and theme explanations made sense to the corresponding author and assistant moderator. Analysis after four groups was compared with that from the final two groups to indicate when sufficient data had been collected. Writing up included illustrative participants’ quotes and discordant data.^46^ Analysis was completed and presented to NHS organizations in Manchester in October 2016. This study was written for publication following publication of the baseline results from the Manchester Lung Screening Pilot in 2018.^31^

## 4 RESULTS

### 4.1 Summary

Fifty‐one participants were recruited; however, 18 (11 smokers; seven ex‐smokers) dropped out leaving 33 participants (11 smokers; 22 ex‐smokers). Groups continued with reduced numbers. Participants included men and women with mixed ages (age range: 50‐80) and socioeconomic backgrounds. Employment status varied; around half were retired. The majority were ex‐smokers and of white ethnicity. There were more male current smokers than female (eight men; three women). Over two‐thirds knew someone who had had cancer, around a third for lung cancer. Demographic characteristics of participants are shown in Table 1 below.

**Table 1:** Participants Demographic characteristics

Participants	Gender (n)	Age (n)	Employment (n)	Cancer experience: not lung cancer^a^ (n)	Cancer experience: Lung cancer^b^ (n)	Ethnicity (n)	Deprivation^c^ (n)
Ex‐Smoking participants							
22	11 men 11 women	1 aged 50‐54 9 aged 55‐64 10 aged 65‐74 1 aged 75‐80 1 unknown	6 employed 11 retired 5 unknown	14 yes 8 no	7 yes 15 no	18 White British 1 White Other 1 South Asian 1 Black/African/Caribbean/Black British 1 Unknown	11 Most deprived 2 Moderately deprived 2 Average 3 Least deprived 4 Unknown
Smoking participants							
11	8 men 3 women	5 aged 55‐64 6 aged 65‐74	0 employed 3 unemployed 6 retired 2 unknown	10 yes 1 no	5 yes 6 no	11 White British	8 Most deprived 0 Moderately deprived 1 Average 2 Least deprived 0 unknown

a Cancer experience: not lung cancer refers to experiences of cancer personally or in family/friends.

b Cancer experience: lung cancer refers to experiences of lung cancer in family/friends.

c Address deprivation has been classified using the Indices of Multiple Deprivation 2015 for Lower Super Output Areas (LSOA) as follows: Most deprived—Address postcode in 10% and 20% most deprived LSOA; Moderately deprived—Address postcode in 30% and 40% most deprived LSOA; Average—Address postcode in 50% most deprived LSOA; Least deprived—Address postcode 60% most deprived LSOA or higher.

While nearly all participants were supportive of lung screening as an idea, many participants expressed a dilemma about whether to be screened if the opportunity arose. Two main themes were found:

1. *Acceptability*: This was about participant's views of lung screening as an idea.
2. *Desire to know about personal lung health*: This was about whether participants wanted to find out about their individual lung health, or not, via screening. Four subthemes were identified:
  - Benefits participants felt they would gain from screening
  - Emotions such as worry about being diagnosed or undergoing tests
  - Practicalities such as service accessibility
  - Smoking including perceived personal smoking risk and stigma.

### 4.2 Theme one: acceptability

This theme is about the acceptability of lung screening. Lung screening as a general idea was found to be widely acceptable and strongly welcomed by nearly all participants. Many participants talked about smoking and coughing as an indication that lung health was generally poor for them, their family or friends. Several recounted experiences of family and friends with lung cancer dying quickly following diagnosis.

Oh, I think it's a very good idea because I have two brothers who ‐ well we all grew up in a family of smokers and beyond that even grandparents and ‐ but ‐ well both my brothers live near x anyway so that would be good and they're still smoking and terrible coughs. In fact their dad, actually their dad died of lung cancer but he'd smoked from 12 to 82 and that wasn't the only thing that was on his death certificate. It was like pneumonia and ‐ I'm not that bothered about myself even though I smoked but listening to them coughing, it's awful, just like their dad, he coughed all his life really and my life. Female6ExsmokerFG2

In this context, lung screening was conceptualized as “an early warning system” (Male1SmokerFG4, Male6SmokerFG6) and a way to stay well in older age. Having your lungs checked via screening was considered a logical approach based on general screening knowledge related to established screening programmes for other diseases rather than lung screening specifically.

It's like the bowel screening programme thing, like that. It's got to be beneficial hasn't it? Male4ExsmokerFG1

Many participants were so enthused by the idea of lung screening that they felt the eligibility criteria, which were generally viewed as cost‐limiting measures, should be broadened to include older and younger people. It was generally accepted that a screening service was needed by smokers and ex‐smokers. Concerns were raised in most groups about excluding others considered to be at risk, such as passive smokers and people exposed to industrial and environmental pollutants.

In terms of broadening the group, first of all, some heavy smokers are diagnosed with lung cancer before the age of 50. So, going further down, so from maybe 45, if it could be afforded it might be a good idea. But the other thing, the other category is passive smokers, because in fact when I said before that one of the two friends I lost through lung cancer recently one of them was a non‐smoker. She had been living with a heavy smoker for all her adult life. So, she almost certainly did it, got it as a result of passive smoking. So you have to ‐ when you look at the risk factors, you have to factor that in. Male10ExSmokerFG3

It's more or less ‐ it's about smoking. What about people let's say who've worked with asbestos and things like that and never smoked in their lives? Male2ExSmokerFG1

### 4.3 Theme two: desire to know about personal lung health

This theme is about whether participants would want to know personally if they had a lung disease via lung screening. Participants talk about whether they wanted to know, or not, about their lung health was threaded through discussions about why they would or would not use a screening service. Four subthemes were identified as follows: screening benefits; emotions; practicalities; and smoking.

Having read a sample invitation letter and accompanying leaflets, participants were asked what they would with do if they received an invite. Participants fell broadly into three groups—the majority expressed a desire to be screened and roughly equal numbers of the others were either undecided or would decline. Those declining included participants who expressed positivity about the lung screening idea. In this sample, the proportion of smokers and ex‐smokers expressing intention to be screened was broadly similar. Making a decision to be screened for some participants appeared quick and straightforward; for others, it was more difficult or tentative. For some participants not knowing about your lung health was considered a better choice than finding out if you had a lung disease. The phrase “head in the sand” (Female7ExsmokerFG2, Female2ExsmokerFG1) depicted this nonparticipation choice.

Head in the sand I think. Yes, it's like this idea of living in ignorance really which isn't good but there is a tendency for a lot of humans to be like that isn't there? Female7ExsmokerFG2

I think there's a huge inertia about getting people to go to these sort of things because they're going to get it [invite letter] and think ooh, do I really want to know? Male3ExsmokerFG1

#### 4.3.1 Subtheme one: lung health check benefits

Participant views about the benefits they could gain from being screened appeared closely linked to their desire to know—or not—about their personal lung health. Many participants, especially ex‐smokers, were worried about smoking damage and felt lung screening could provide them reassurance. For these participants, “peace of mind” (Female2ExsmokerFG1; Male5SmokerFG5 and Female3SmokerFG5) was a key benefit motivating screening uptake.

Well it gives you a health check basically so it's good to be reassured that your lungs are okay or it's good to know there's something wrong and you need to seek further advice and get something done about it. Male4Exsmoker FG1

Many participants felt that quick detection could result in a better outcome if lung cancer was found. Personal experiences around other cancers where early diagnosis had positive outcomes were recounted in most groups. Participants who considered lung cancer treatable if found early were generally keen to have their lungs screened. Screening harms such as radiation risks were discussed by a minority in one group only and considered as outweighed by the early treatment opportunity.

See my outlook has really changed since I've been through ‐ over the last 5 years or so, it's made my change because I know that if I hadn't done something about it I wouldn't be here. Well I wouldn't probably have given it what's it but if I hadn't have done because a mate of mine had the same thing…[Over speaking]…and he was what's it ‐ that I went and it changed my outlook altogether. Male2SmokerFG4

However, several participants were concerned that screening might result in a serious lung cancer diagnosis where treatment options were limited. For these participants, the opportunity to find out about lung health did not offer much personal benefit. Some current smokers from deprived areas had particularly low lung and general health expectations and expressed fatalistic beliefs.

I know people that have been asked to go for check‐ups and this, that and the other and they think they're going to live forever. They're only 42. When you get into 55, you're sort of thinking hang of a minute, I've only got 10 years to go. Male6SmokerFG6

Correct. Not to retire, to lean over. Male7SmokerFG6

For a few participants, potential lung cancer symptoms were viewed as a trigger point for screening uptake even though screening was aimed at nonsymptomatic individuals. For one participant, with other diagnosed health conditions, screening was viewed negatively due to higher concern about existing conditions.

I wouldn't go and expect tests done if I didn't have any signs or anything. Female11ExSmokerFG3

I've already got multiple things to deal with medically which are very stressful and burdensome. Do I want another thing? Can I handle it? Maybe not. Not at my age. So I would just leave it because I would also think that it might not be very important in thinking of what I already am going through. Female4ExSmokerFG1

#### 4.3.2 Subtheme two: emotions

It was clear from participant's discussions that lung cancer screening was an emotive topic. Anxiety was expressed about being diagnosed with lung cancer following screening and about the screening process itself. While many participants expressed some worry about smoking damage which motivated uptake, for others the fear of a cancer diagnosis loomed so large that they could not face finding out about their lung health. Some participants relayed stories of ignoring potential cancer symptoms or nonparticipation in screening programmes due to fear.

It swelled up and so I'll maybe have that checked out. I said, it'll be all right and I never mentioned it again but it grew and grew and I used to wear a polo neck to cover it because I thought it's a cancer lump because it went to about the size of my fist. Then my daughter was born and I thought I'll have to have this done for the sake of my daughter. Because I was terrified of all hospitals, anything to do with hospitals, you could forget it, I'd rather suffer than go in if you know what I mean? Male1SmokerFG4

Several participants expressed anxiety about the process of screening—attending hospital, undergoing screening tests and waiting for results. The thought of interaction with doctors and medical settings generated anxiety for several participants. Many participants expressed anxiety about LDCT scans due to confusion with magnetic resonance imaging scanning and claustrophobia; a few were concerned about spirometry.

The tunnel puts me off. I'm claustrophobic and that certainly doesn't ‐ I mean I'd still do it but I'd be petrified. Male1ExsmokerFG1

But you do start to panic when you read like ‐ and you can't breathe for 10 seconds. In your head you make 10 seconds into 10 minutes. Female7ExsmokerFG2

The Manchester one‐stop community‐based lung health check, looking for a range of lung diseases not just cancer, seemed to help reduce anxiety and increase positivity towards uptake. Just one participant suggested that leaving a little time between the health check and a follow‐up scan (if required) might be preferable.

I think that's the frightening thing for people if you think, oh, they're just looking for cancer. Well that tells you on here that we're not, we're just looking for anything to do with your lungs. Because I think saying ‐ and you think it's not just about cancer it's about your general lung health is a good thing. Female5ExsmokerFG2

I'd prefer to go straight on, otherwise you go home and panic and you get yourself worse, so you're better getting it done all at once. Female11ExSmokerFG3

When you're told that maybe there's something wrong, you might want to step back a bit and just ‐ because it could be a little bit of a shock to you and you think oh God, am I going to die next week sort of thing. So maybe you want to go home and think about it. Female10ExSmokerFG3

#### 4.3.3 Subtheme three: practicalities

Simple practicalities (location, booking speed and appointment availability) were identified as barriers to lung screening uptake by many participants. For participants with positive, but tentative uptake intentions, practical barriers would discourage uptake. A community‐based screening service was viewed positively for convenience, and some participants suggested that this would increase the likelihood of them being screened.

I might just ring, just see if they're engaged. If they're engaged, forget it. Then I won't ring back. Male7SmokerFG6

Where they're going to put this mobile what's it? Say it was on the supermarket and you were doing your shopping and you saw that, if you could go in and get it done. Male3SmokerFG4

#### 4.3.4 Subtheme four: smoking

Smoking, including stigma and smoking risk, was discussed in all groups. For many, smoking was the key reason why they felt they would benefit from screening. However, for others smoking was a participation barrier. While most participants recognized a link between smoking and lung cancer, several, especially current smokers, talked extensively about pollutants causing lung cancer and other's need for screening (due to coughing or heavier smoking) in ways which suggested limited recognition or acknowledgement of personal smoking‐related cancer risk.

So being healthy isn't going to make sure you're going to be healthy when you get older. Because it's not just smoking what gives you lung cancer. Male7SmokerFG6

Well we live in a bad area don't we? I mean I grew up when cotton mills were belching out smoke all over the place, you couldn't look into my village X when I grew up. You went up on the moors and looked down and it was in a fog. That's what I grew up you know so ‐ and I think yeah, you know Glasgow and there's a lot of background radiation in Glasgow isn't there from the granite. Male3SmokerFG4

Many participants talked about screening as revealing the personal impact of smoking and one suggested screening uptake required taking personal responsibly for a health‐damaging habit. A few participants suggested that as smokers, they would be treated less sympathetically by medical professionals should lung cancer be found.

It's you, it's a personal ‐ why, the consequence of you smoking. But you know that every time you took a cigarette out the packet; you knew it's detrimental to your health. So now we're going to find out proper, knock on the head, it was you and all them years taking responsibility for your consequences. Female2ExsmokerFG1

I think that if you're a smoker or an ex‐smoker a lot of doctors treat you like you're a leper. It's a dirty disease because you smoke. Female5ExsmokerFG2

For some, mainly ex‐smokers, lung screening was viewed as part of pursuing healthier habits. Smoking cessation was not extensively discussed; however, a few current smokers indicated a diagnosis would prompt quitting, and others felt an all‐clear would suggest they still had chance to improve their lung health through quitting.

## 5 DISCUSSION

### 5.1 Multifaceted decision making

There were multiple influences on participants’ uptake decision‐making intentions. This aligns with others findings^18^, ^19^, ^20^ as did the reasons why some participants expressed a preference for screening (early diagnosis and potential reassurance) and some did not (lung cancer diagnosis fear and low levels of perceived personal benefit).^17^, ^18^, ^19^, ^22^, ^29^ Other studies have also found that waiting for results and hospital attendance created anxiety^19^, ^21^, ^30^ and were participation barriers.^18^, ^21^ However, there were differences with some of the literature. In this study, while some participants expressed fatalism and did not feel they would benefit due to low lung health expectations and limited belief in treatment effectiveness, this was not as widespread as indicated for London's deprived area smokers.^19^ Many participants in our study expressed keenness to be screened as this could result in finding lung cancer early and increase survival chances. This is more in line with Cataldo's findings^30^ and may relate to this study's more socioeconomically mixed sample or it is possible that as the researchers were NHS employed with study participation invitations arising from an NHS organization this may have reduced negative comments. This was mitigated through building rapport with participants, the topic guide and seeking alternative views; however, the researcher's influence cannot be discounted.^47^ A further difference to the London study was in the anxiety expressed about screening tests, including the LDCT scan due to claustrophobia. Fear of having a CT scan was reported by Cataldo^30^ and fear of subsequent treatment by Carter‐Harris et al.^22^

### 5.2 Emotional barriers

In our study, the preference not to be screened was strongly linked to a fear of being diagnosed with lung cancer as has been found elsewhere.^18^, ^19^, ^21^, ^22^ Cancer worry was reported as an uptake barrier in the UKLS for current smokers, women and people from more deprived areas where widespread negative experiences of lung cancer may generate more pessimistic attitudes.^21^, ^48^ In this study, we also found that anxiety about personal disease risk motivated screening uptake for some participants. The desire for “reassurance” via lung screening has been found elsewhere^17^, ^18^, ^19^ including in other screening programmes where worry has been reported as motivational for some and a barrier for others.^25^ In view of the influence of worry on lung screening uptake, the communication of survivorship stories, information about effective treatments and screening tests may help reduce fear barriers.

### 5.3 Smoking

In this study, similar proportions of smokers and ex‐smokers expressed positive screening intent in response to viewing sample invitational materials. However, some differences were indicated. When asked what they would do with an invite letter, ex‐smokers expressed more definite positive screening intentions than current smokers whose views appeared more tentative. Current smokers also commonly talked about how other's needed screening more than they did, due heavier smoking or worse symptoms and emphasized pollution‐related lung cancer causation. This suggested personal smoking risks and screening need was being downplayed. However, some of these same participants were also extremely worried about a cancer diagnosis. This was so high for some, especially current smokers, that they felt cancer would almost inevitably be found if they were screened—as has been reported elsewhere.^19^ This seeming contradiction supports Quaife et al's^49^view that smokers experience “cognitive dissonance”^50^ about continuing to smoke while knowing its risks. It has been suggested that this results in biased thought processing^51^, ^52^, ^53^, ^54^, ^55^ distorting risks in “defensive denial”,^56^ not wanting to be faced with risk information, such as would be apparent from attending lung screening.^17^, ^18^, ^19^, ^22^, ^49^, ^57^ The indication in this study that lung screening uptake required facing‐up to smoking consequences supports this. In fact, “avoiders” were one of Patel's nonresponder types.^18^ However, this finding is tentatively presented as there were fewer current smokers (11 smokers; 22 ex‐smokers) and a lower number of female current smokers (three women; eight men) than planned due to participant drop out which limits these findings. We also need to consider that smokers are not a homogenous group and lung screening views may also vary with socioeconomic status^28^ and other factors. For example, Hahn^58^ reported that screening interest varies with quit intention with smokers actively considering quitting more interested than others.

### 5.4 An Informed choice?

Screening participation should be an “informed choice”,^59^, ^60^, ^61^, ^62^, ^63^ which is a decision made with understanding about the disease being screened for, possible treatments, harms and benefits and in alignment with personal views.^64^ However, around half of NELSON trial invitees (a Dutch Belgium lung screening randomized control trial) were reported to have insufficient knowledge of disease, personal threat and screening processes to make well‐founded choices with lower knowledge levels about lung screening in nonparticipants than participants.^20^ In our study, several participants appeared to have limited knowledge or misunderstandings about the optimal time to be screened (before symptoms were present), early diagnosis benefits, screening relevant concepts (such as risk thresholds and smoking pack‐years) and some screening tests. Participant knowledge came mainly from general cancer screening information rather than that specific to lung cancer screening. Similar misunderstandings and knowledge gaps have also been found in the United States.^17^ Our study's participants commented that a decision to participate, or not, in lung screening was their choice, but to be an “informed choice,” improved participant knowledge is needed.^17^, ^19^, ^62^, ^63^

In the United States, joint discussions between patients and health care providers have been mandated by Medicare in the lung screening process to support informed decision making.^64^ Supporting informed and equitable lung screening uptake is an anticipated challenge in the UK.^65^, ^66^ Decisions about screening uptake require weighing up complicated information^8^, ^67^ and a high degree of “health literacy”,^68^, ^69^, ^70^, ^71^ the comprehension of health information in order to make a well‐founded choice. However, 43% of UK working age adults have been found to have difficulties understanding written health information; 61% when numerical data are included.^72^ Poor levels of health literacy found have also been found in older UK smokers,^73^ the key target group for lung screening. In the United States, some decision aids have been developed and tested with promising results for supporting decision making^66^, ^68^, ^69^, ^70^, ^71^, ^74^, ^75^, ^76^, ^77^, ^78^; however, the readability of online aids has been challenged^79^, ^80^ and further testing is needed.^76^, ^81^ In the UK, there are currently no guidelines for developing quality assured lung screening patient literature or established decision‐making tools. Future research could usefully consider the feasibility and impact of lung screening decision aids in a UK context.

### 5.5 Push and pull in decision making

**Figure 1:**
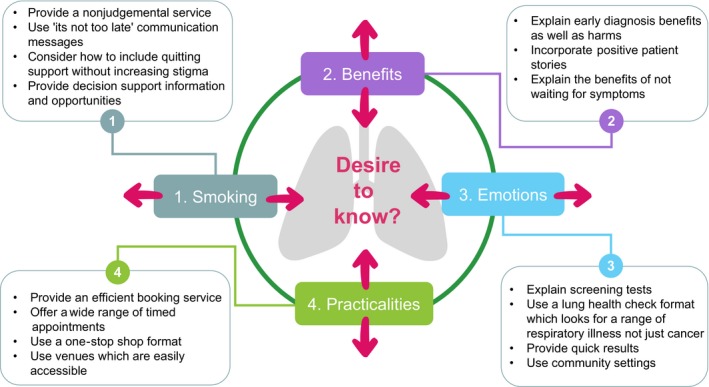
“Push and pull” in the desire to know about lung health with lung screening practice recommendations

The desire to know about personal lung health in this study was influenced by participant's views about benefits; emotions; smoking; and practicalities. These appeared to motivate uptake for some participants but were a barrier for others. For example, the anticipation that a lung health check would result in personal health benefit encouraged uptake, but low benefit expectations discouraged attendance. Worry could either encourage lung screening or was an obstacle. Practicalities about service access—their ease or difficulty acted similarly. Smoking was a barrier to uptake where health risks were downplayed or where stigma was felt, but was motivational when risks were recognized and faced. Figure 1 below illustrates this “push and pull” effect on uptake decision‐making intentions pictorially. This is important as it indicates points where public health action and careful service design could minimize participation barriers. Practice‐based actions are suggested in Figure 1 relating to the theme “desire to know about personal lung health”. This draws on the experience of the Manchester NHS lung screening pilot which has recently reported its baseline findings.^31^

### 5.6 Study strengths and limitations

Our study is useful to build understanding as lung screening pilots are now being developed in the NHS^31^ and adds to the limited UK evidence base outside of a trial context. However, these findings should be considered limited by sample make‐up and context^47^ in this case a postindustrial city^37^ with high incidence lung cancer and deprivation.^38^, ^82^ Given the relatively small purposive sample, it is also possible that participants may have different views to the wider population and that invitations from an NHS organization to participate may have reduced expression of some negative screening opinions. Focus group discussions sparked conversations revealing insights but may have concentrated opinions.^83^ Alternative views were sought, discordant data included and separate groups for current and ex‐smokers allowed free expression of views. The final two groups did not produce additional themes, but confirmed those found previously.

## 6 CONCLUSION

This study suggests that lung screening was widely acceptable and welcomed by participants with similar characteristics to the screening target group. The desire to know about personal lung health was influenced by emotions; benefit views; practicalities; and smoking. Current smokers appeared to have higher uptake barriers than ex‐smokers and tackling this will be a key challenge in lung screening programmes. Some barriers can be addressed at the service level for which recommendations have been made. Future research could helpfully explore the effectiveness of different types of informational materials in supporting “informed participation” and the views of smokers further. Lung health checks have the potential to open up early treatment benefits to a high cancer risk group and reduce health inequalities. However, this will only be possible if consideration is given to reducing the identified barriers in future lung screening programmes.

## CONFLICTS OF INTEREST

None declared.
